# Absence of EPAC1 Signaling to Stabilize CFTR in Intestinal Organoids

**DOI:** 10.3390/cells11152295

**Published:** 2022-07-25

**Authors:** João F. Ferreira, Iris A. L. Silva, Hugo M. Botelho, Margarida D. Amaral, Carlos M. Farinha

**Affiliations:** BioISI-Biosystems and Integrative Sciences Institute, Faculty of Sciences, University of Lisboa, Campo Grande, 1749-016 Lisboa, Portugal; jfsferreira@fc.ul.pt (J.F.F.); iasilva@fc.ul.pt (I.A.L.S.); hmbotelho@fc.ul.pt (H.M.B.); msamaral@fc.ul.pt (M.D.A.)

**Keywords:** CFTR, Cystic Fibrosis, cAMP signaling, EPAC1, membrane stability, intestinal organoids

## Abstract

The plasma membrane (PM) stability of the cystic fibrosis transmembrane conductance regulator (CFTR), the protein which when mutated causes Cystic Fibrosis (CF), relies on multiple interaction partners that connect CFTR to signaling pathways, including cAMP signaling. It was previously shown that activation of exchange protein directly activated by cAMP 1 (EPAC1) by cAMP promotes an increase in CFTR PM levels in airway epithelial cells. However, the relevance of this pathway in other tissues, particularly the intestinal tissue, remains uncharacterized. Here, we used Western blot and forskolin-induced swelling assay to demonstrate that the EPAC1 protein is not expressed in the intestinal organoid model, and consequently the EPAC1 stabilization pathway is not in place. On the other hand, using cell surface biotinylation, EPAC1-mediated stabilization of PM CFTR is observed in intestinal cell lines. These results indicate that the EPAC1 stabilization pathway also occurs in intestinal cells and is a potential target for the development of novel combinatorial therapies for treatment of CF.

## 1. Introduction

The cystic fibrosis transmembrane conductance regulator (CFTR) protein is a member of the ATP-binding cassette (ABC) transporter superfamily [1]. Mutations in the *CFTR* gene lead to Cystic Fibrosis (CF)—the most common lethal and life-limiting autosomic recessive disease among the Caucasian population [2]. CF is mostly characterized by airway disease, pancreatic insufficiency, salt concentration increase in sweat and male infertility [3]. Among the more than 2100 variants described, F508del is the most prevalent mutation, occurring in ~80 % of individuals with CF worldwide [1].

The balance between anterograde transport, endocytosis, and recycling is responsible for the regulation of CFTR steady-state levels at the plasma membrane (PM) [4,5]. These processes rely on the interaction of CFTR with several protein partners [5,6], actin cytoskeleton integrity and cAMP signaling [7]. A two-level regulation of CFTR by cAMP has been proposed. Low concentrations of cAMP activate protein kinase A (PKA), which phosphorylates the regulatory domain of CFTR activating channel function [1]. On the other hand, in bronchial epithelial cells higher concentrations of cAMP lead to activation of the exchange protein directly activated by cAMP 1 (EPAC1), triggering translocation from the cytoplasm to the PM [8], where it interacts with CFTR via the adaptor protein Na^+^/H^+^ exchanger regulatory factor 1 (NHERF1) leading to an increase in the amount of CFTR at the PM, due to decreased endocytosis [9]. More recently it was shown that this stabilizing process involves the recruitment of several actin cytoskeleton regulators to the CFTR vicinity, namely the proteins inverted formin 2 (INF2) and F-actin-capping protein subunit alpha-2 (CAPZA2) [10]. While PKA and EPAC1, individually, participate in these regulatory complexes, in general, their signaling complexes are non-overlapping [11].

EPAC1 is a guanine nucleotide exchange factor (GEF) for small Ras-like GTPases that belong to the Ras superfamily of small G proteins, Rap1 and Rap2 [12]. As a GEF, EPAC1 catalyzes the exchange of GDP for GTP and thereby controls the activation of Rap-mediated processes and suppresses Ras-mediated oncogenic cell transformation [13].

The role of EPAC1 in stabilizing CFTR at the PM has been addressed in an airway epithelial cell model [9]. However, there is no information on the relevance of the EPAC1 stabilization pathway on other tissues including intestinal cells and, in particular, intestinal organoids. Herein, we explore the EPAC1-mediated stabilization of CFTR at the PM in intestinal models, including intestinal organoids. We show that EPAC1 activation does not increase intestinal organoid swelling due to absence of protein expression. We also show that EPAC1 is expressed in intestinal cell lines (Caco-2 and NCM460) and that its activation promotes the increase in CFTR PM levels. Altogether, these results prove that EPAC1 activation is relevant in stabilizing PM CFTR, even though the pathway does not occur in intestinal organoids.

## 2. Materials and Methods

### 2.1. Cell Culture and Compound Treatment

Cystic Fibrosis Bronchial Epithelial (CFBE41o-) cells [14], referred to as CFBE, stably transduced with wt-CFTR [15] and Caco-2 cells were cultured in MEM (Corning Inc., Corning, NY, USA, 10-010-CV) supplemented with 10% (*v*/*v*) FBS (Gibco, Waltham, MA, USA, 10270-106) and, only for CFBE cells, 2.5 μg/mL puromycin (Sigma, Burlington, MA, USA, P8833). NCM460 cells were cultured in RPMI (Corning, 10-040-CV) supplemented with 10% (*v*/*v*) FBS. All cell lines were maintained at 37 °C in 5% (*v*/*v*) CO_2_. Passage numbers were kept at the minimum possible never exceeding 35.

When applicable, cells were treated for 24 h with 5 μM of CFTR corrector VX-661 (Selleckchem, Houston, TX, USA, S7059) in the appropriate medium without FBS. Furthermore, cells were treated for 2 h or 3 h with varying concentrations (1, 3, 5 or 10 μM) of 007-AM (BioLog, Bremen, Germany, c051) or vehicle control (0.1% DMSO *v*/*v*) in the appropriate medium without FBS. All compound treatment conditions are in accordance with standardized procedures previously used [9,16].

### 2.2. Forskolin-Induced Swelling Assay

Intestinal organoids were available after crypt isolation from rectal biopsies of individuals with CF, according to [17]. Rectal biopsies were obtained from individuals with CF upon Ethical approval by the hospital Ethical committee. Intestinal organoids from individuals with D1152H/F508del-CFTR genotype were seeded in a pre-warmed 96-well plate and FIS assay was performed as previously described [18]. When applicable, intestinal organoids were treated with 5 μM VX-661 for 24 h and 3, 5, or 10 μM 007-AM for 3 h before the assay, both prepared in Advanced Dulbecco’s Modified Eagle Medium/Ham’s F-12. Then, 30 min before the assay, organoids were incubated with calcein green (8 μM, Invitrogen, Waltham, MA, USA, C34852). After calcein green staining, forskolin (Fsk; Sigma, F6886) was added at different concentrations (0.02, 0.128, 0.8 or 5 μM). Live cell imaging was performed on a Leica SP8 confocal microscope (Leica Microsystems, Wetzlar, Germany) for 60 min at 37 °C, with image acquisition every 10 min. Quantification was carried out as previously described [19], using CellProfiler software [20] and the area under the curve (AUC; t = 60 min, baseline = 100%) was calculated using GraphPad Prism software (GraphPad, Waltham, MA, USA).

### 2.3. Plasmid Transfection

For cell transfection, a mixture containing 0.2 μg of green fluorescent protein (GFP)-EPAC1 plasmid DNA and 0.5 μL of Lipofectamine 2000 in 50 μL of OptiMEM (Gibco, 31985062) was prepared according to the manufacturer’s instructions. The mixture was added to cells 24 h after splitting. After 24 h, transfection mixture was removed, and medium changed to MEM supplemented with 10% (*v*/*v*) FBS.

### 2.4. Live Cell Imaging

Fluorescence imaging was performed 24 h after transfection of cells with GFP-EPAC1 plasmid. Cells were maintained at 37 °C in PBS and imaged on a Leica SP8 inverted confocal microscope equipped with a 20 × 0.75 NA dry objective. Excitation was performed with a solid state 488 nm laser. Detection was performed by setting the pinhole at 1 airy unit, using a photomultiplier tube detector, and selecting an adequate emission band. The intracellular localization of EPAC1 in live cells was assessed by monitoring GFP fluorescence signal for 18 min with images acquired every 3 min. After the 3 min image acquisition, cells were stimulated with 1 μM 007-AM or vehicle control (0.1% DMSO) in PBS. Images were acquired using Leica Application Suite X software (Leica Microsystems, Germany) and for fluorescence pixel intensity quantification in a region of interest on single optical slices, Fiji–ImageJ software was used.

### 2.5. Western Blotting

For protein extraction, organoids and cells were washed three times with cold PBS and lysed with the appropriate volume of sample buffer. Protein samples were separated by SDS polyacrylamide gel electrophoresis using a 10% separating gel and a 4% stacking gel. Samples run on Tris-Glycine-SDS buffer (BioRad, Hercules, CA, USA, 1610772) at 60 V when in the stacking gel and 120 to 140 V when in the separating gel during the time needed to resolve the molecular weight range of interest. Next, proteins were transferred onto polyvinylidene difluoride membranes (Merck, Kenilworth, NJ, USA, PR04574) using Tris-Glycine buffer (BioRad, 1610771) at 400 mA for 90 min. After transfer, membranes were blocked for 1 h with 5% (*w*/*v*) skimmed milk in PBS supplemented with 0.1% (*v*/*v*) Tween 20 (PBS-T; Thermo Fisher, Waltham, MA, USA, BP337) and then incubated overnight at 4 °C with primary antibodies diluted in 5% (*w*/*v*) skimmed milk in PBS-T (1:3000 mouse anti-CFTR 596 (Cystic Fibrosis Foundation, A4), 1:1000 rabbit polyclonal RAPGEF3 antibody—middle region (Aviva Systems Biology, San Diego, CA, USA, ARP52140_P050), 1:3000 purified mouse anti-ezrin (BD Transduction Laboratories, Franklin Lakes, NJ, USA, 610602)). α-tubulin (1:10,000 mouse monoclonal anti-α-tubulin1 (Sigma, T5168)) was detected as loading control. After washing with PBS-T, membranes were incubated with the adequate secondary antibody diluted in 5% (*w*/*v*) skimmed milk in PBS-T for 1 h at room temperature (1:3000 goat anti-mouse IgG (H + L) HRP conjugate (Bio-Rad, 170-6516) or 1:3000 goat anti-rabbit IgG (H + L) HRP conjugate (Bio-Rad, 170-6515)). Lastly, membranes were washed again with PBS-T. The Clarity Western ECL substrate (BioRad, 170-5061) was used with Chemidoc XRS plus analyzer (BioRad) for chemiluminescent detection. The ImageLab software (BioRad) was used for band intensity quantification, which is represented after normalization to the corresponding loading control.

### 2.6. Cell Surface Biotinylation Assay

Cell surface biotinylation was performed as previously described [21]. Briefly, cells were washed three times for 2 min with cold PBS^++^ (PBS pH 8.2; 1 mM MgCl_2_; 0.1 mM CaCl_2_) and then incubated at 4 °C for 30 min with 0.5 mg/mL of membrane impermeable Sulfo-NHS-SS-Biotin (Thermo Fisher, 21331) in PBS^++^. After incubation cells were washed three times for 2 min with cold PBS^++^ and lysed with the appropriate volume of BL buffer (25 mM HEPES (Sigma, H3375) pH 8.2; 1% (*v*/*v*) Triton X-100 (Sigma, T9284); 10% (*v*/*v*) glycerol and supplemented with complete protease inhibitor cocktail) for 15 min at 4 °C. After cell scrapping to collect lysates, centrifugation was performed at 14,000× *g* for 10 min at 4 °C. A portion of the supernatant was incubated with sample buffer supplemented with 100 mM DTT (Sigma, D0632) for 30 min at 37 °C to collect the whole-cell lysate (WCL) sample. The remaining supernatant was incubated overnight at 4 °C with streptavidin beads (Sigma, S1638), pre-washed with PBS^++^ and BL buffer. The next day, centrifugation was performed at maximum speed for 1 min at 4 °C and then beads were washed three times with BL buffer. Next, the beads were incubated with sample buffer supplemented with 100 mM DTT for 5 min at 85 °C and, after pulse spin, the pull-down sample (biotinylated proteins) was collected. Both samples collected were analyzed by Western blot (WB).

### 2.7. Statistical Analysis

Data are presented as a mean and standard error of the mean (SEM) and were analyzed using two-tailed Student’s *t*-tests for unpaired samples with *p* < 0.05 considered as the level of statistical significance.

## 3. Results

### 3.1. Activation of EPAC1 by AM-007 Promotes Its Translocation to the PM in CFBE

The study of EPAC1-mediated events is facilitated by compounds that selectively activate it, independently from PKA. As EPAC1 lacks the glutamate residue that in PKA interacts with the ribose of cAMP, a selective EPAC1 agonist was developed, 8-pCPT-2′-O-Me-cAMP, and an acetoxymethyl (AM)-ester was introduced to increase cell permeability. This compound (known as 007-AM) is ten times more efficient than cAMP in activating EPAC1 [22,23,24].

**Figure 1:**
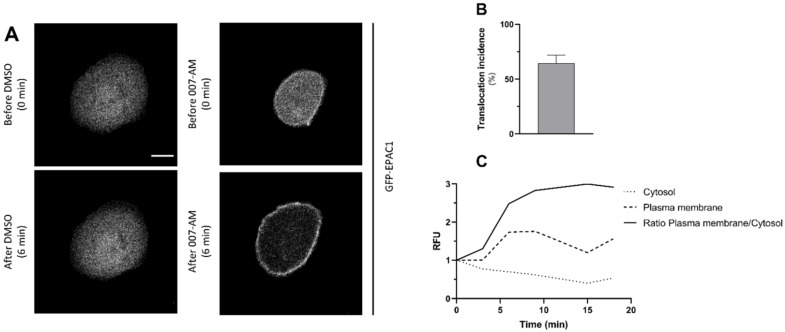
EPAC1 translocation after treatment with 007-AM in CFBE cells. (**A**) Localization of GFP-EPAC1 was determined by confocal live-cell imaging in CFBE wt-CFTR cells. Panels display detection of EPAC1 before (upper panels) and 6 min after (lower panels) addition of 1 μM 007-AM (right panels) or DMSO (left panels). Scale bars: 10 μm. (**B**) Quantification of the fraction of cells that display EPAC1 translocation to the PM after 007-AM stimulation (counting was carried out as described [9]; *n* = 3, 10–15 cells per experiment). (**C**) Representative intensity of pixel fluorescence (relative fluorescence units, RFU) of GFP–EPAC1 at the PM versus in the cytoplasm during stimulation with 1 μM 007-AM relative to pre-stimulus levels in CFBE wt-CFTR cells. Image acquisition was performed every 3 min and 007-AM stimulation was initiated at 3 min.

We started by confirming the ability of our batch of AM-007 to activate EPAC1. For this, an EPAC1 translocation assay was performed using CFBE wt-CFTR cells (since after activation, EPAC1 is targeted from the cytoplasm to the PM). First, the cells were transfected with the GFP-EPAC1 cDNA plasmid so that EPAC1 localization could be monitored through the GFP fluorescence signal. Then, live-cell imaging was performed, and after a 3 min baseline period cells were stimulated with 007-AM to activate EPAC1, or DMSO (vehicle control) (Figure 1).

The results of live-cell imaging show that, under basal conditions, EPAC1 is mainly localized in the cytoplasm (Figure 1A, upper panels). After activation by 007-AM, EPAC1 translocates to the PM and this occurs within the first 3 min of stimulation with the cAMP analogue (Figure 1A, bottom right panel; Figure 1C) in around 64% of the cells (Figure 1B). This translocation is not observed in the negative control under DMSO treatment (Figure 1A, bottom left panel). Translocation stabilized after 6 min of 007-AM stimulation, when 75% of the EPAC1 pool was found associated with the PM (Figure 1C). The results obtained indicate that 007-AM activates EPAC1 promoting its translocation to the PM in this respiratory cell line.

### 3.2. EPAC1 Activation Does Not Increase Intestinal Organoid Swelling

To assess whether the EPAC1-mediated CFTR stabilization also occurs in intestinal organoids (and thus whether this model can be used to study the EPAC1 pathway in materials derived from individuals with CF), we used the FIS assay, considering that levels of CFTR function detected are dependent not only on the gating of the channel but also on the amount of protein at the PM. As wt-CFTR organoids cannot be used in FIS assays due to organoid pre-swelling before the assay [17], the genotype D1152H/F508del-CFTR was chosen. D1152H is a class IV mutation that has high residual function [25], but without causing organoid pre-swelling and thus allowing the detection of a potential increase in CFTR function.

**Figure 2:**
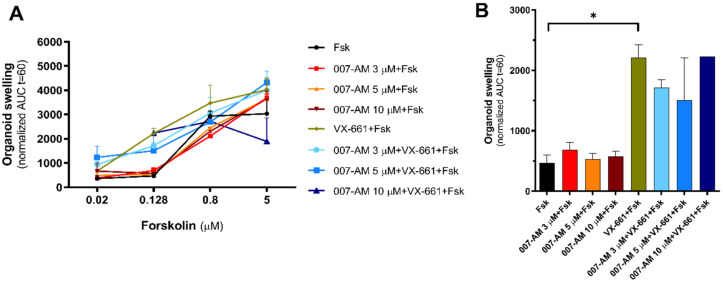
Impact of EPAC1 activation in intestinal organoids. (**A**) FIS quantification in D1152H/F508del-CFTR organoids after treatment with VX-661 (5 μM, 24 h) or 007-AM (3, 5, 10 μM, 3 h), both individually and in combination, at Fsk concentrations of 0.02, 0.128, 0.8 and 5 μM, expressed as the AUC of organoid surface area increase (baseline = 100%, t = 60 min). (**B**) Quantification of organoid swelling at 0.128 μM Fsk for D1152H/F508del-CFTR organoids. Data are shown as the mean of measurements on 1–2 wells per condition ± SEM. Statistical analysis was performed using two-tailed unpaired Student’s *t*-test. * *p* < 0.05 was considered as significant.

Before the FIS assay, intestinal organoids were incubated with the CFTR corrector VX-661 to rescue mutant CFTR and with multiple concentrations of 007-AM both individually and in combination, to activate EPAC1. For the assay, CFTR was stimulated with increasing concentrations of Fsk, and treatment efficacy was analyzed at 0.128 μM Fsk as this is the concentration where, reportedly, correlation between treatment effect and clinical benefit is best (Figure 2) [26,27]. In FIS assays, the AUC corresponds to the area under the curve (AUC) that represents normalized swelling over time, thus being proportional to CFTR function [28].

Results show, as expected, high residual function of the D1152H mutation as observed by the increase in AUC upon stimulation with Fsk without any further treatment (Figure 2A, black line). Results also show that treatment with VX-661 leads to an increase in swelling (Figure 2B, green bar) but EPAC1 activation, with or without CFTR rescue by VX-661, does not further increase organoid swelling under 0.128 μM Fsk stimulation (Figure 2B). These results indicate that EPAC1 activation does not lead to an increase in CFTR function in intestinal organoids, suggesting that the EPAC1 pathway is not active in this model.

### 3.3. EPAC1 Expression Is Not Detected in Intestinal Organoids

**Figure 3:**
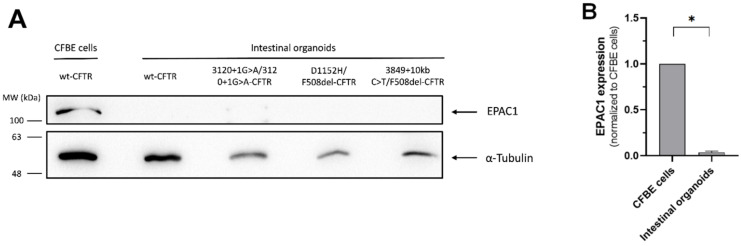
EPAC1 expression in intestinal organoids. (**A**) WB analysis of EPAC1 levels in CFBE wt-CFTR cells and wt-, 3120+1G>A/3120+1G>A-, D1152H/F508del- and 3849+10kb C>T/F508del-CFTR intestinal organoids. For loading control α-Tubulin was detected. (**B**) Quantification of EPAC1 expression levels in intestinal organoids normalized to CFBE wt-CFTR cells. Data are shown as the mean ± SEM, *n* = 4. Statistical analysis was performed using two-tailed unpaired Student’s *t*-test. * *p* < 0.05 was considered as significant.

Given the absence of response to 007-AM, next, EPAC1 expression in intestinal organoids was assessed, as there are no reports of analysis of EPAC1 expression in this model. For this, wt-, 3120+1G>A/3120+1G>A-, D1152H/F508del- and 3849+10kb C>T/F508del- CFTR intestinal organoids were used. 3120+1G>A is a class V mutation [29] that has no residual function, while 3849+10kb C>T is also a class V mutation [29] but with some residual function. The four different intestinal organoid genotypes were chosen to determine if EPAC1 expression is affected by CFTR genotype. Protein was extracted from the organoids and EPAC1 expression levels were assessed by WB in comparison to protein levels in CFBE wt-CFTR cells (Figure 3).

WB results show that EPAC1 levels in intestinal organoids are almost undetectable (Figure 3A) and thus are statistically significantly lower in comparison to CFBE wt-CFTR cells (Figure 3B). The results also show that EPAC1 expression is similar across the four genotypes tested, suggesting that it is independent of CFTR genotype (Figure 3A). These results explain the absence of effect in CFTR function after 007-AM treatment observed in the FIS assays and indicate that the cAMP signaling pathway that stabilizes CFTR at the PM through EPAC1 activation is not active in intestinal organoids due to the lack of expression of EPAC1.

### 3.4. EPAC1 Is Expressed in Intestinal Cell Lines

**Figure 4:**
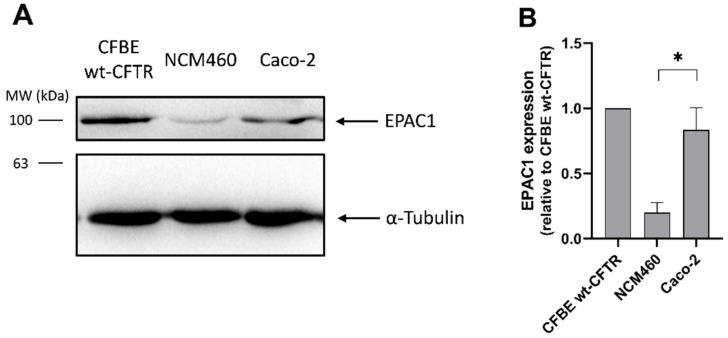
EPAC1 expression in intestinal cell lines. (**A**) WB analysis of EPAC1 levels in CFBE wt-CFTR cells, NCM460 and Caco-2 intestinal cell lines. For loading control α-Tubulin was detected. (**B**) Quantification of EPAC1 expression levels in intestinal cell lines normalized to CFBE wt-CFTR cells. Data are shown as the mean ± SEM, *n* = 3. Statistical analysis was performed using two-tailed unpaired Student’s *t*-test. * *p* < 0.05 was considered as significant.

To understand whether the lack of EPAC1 expression is a general characteristic of intestinal models or it is restricted to intestinal organoids, EPAC1 expression was assessed and quantified in the intestinal cell lines Caco-2 and NCM460. As previously, protein was extracted and EPAC1 expression levels were assessed by WB in comparison to CFBE wt-CFTR cells (Figure 4).

The results show that EPAC1 expression is detected in both intestinal cell lines (Figure 4A), contrary to the findings in the intestinal organoids. There is, however, a statistically significant difference in EPAC1 expression levels between the two cell lines, with NCM460 cells having only around 25% and Caco-2 cells similar levels of EPAC1 expression levels when compared to CFBE wt-CFTR cells (Figure 4B).

### 3.5. EPAC1 Activation Increases PM CFTR in Intestinal Organoids

**Figure 5:**
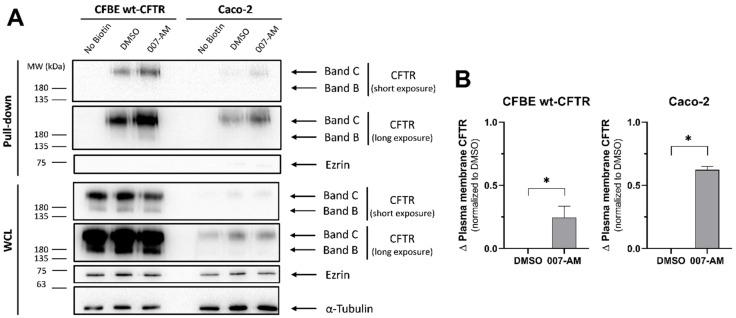
Impact of EPAC1 activation on wt-CFTR levels at the PM in intestinal cell lines. Cell surface biotinylation was performed in CFBE wt-CFTR cells, and Caco-2 cells treated with 007-AM (2 h, 1 μM) or DMSO (vehicle control). Cells were incubated with membrane non-permeant biotin, followed by lysis and overnight incubation with streptavidin beads. As negative control, cells not incubated with biotin were used. (**A**) Detection of CFTR by WB after pull-down with streptavidin. Part of the total lysate was analyzed as WCL. For loading control α-Tubulin was detected and as an intracellular protein control in the pull-down fraction ezrin was detected. To allow better comparison, CFTR blots are shown at both a shorter and a longer exposure. (**B**) CFTR was quantified after the pull-down and the change (Δ) normalized to DMSO was plotted. Data are shown as the mean ± SEM, *n* = 3. Statistical analysis was performed using two-tailed unpaired Student’s *t*-test. * *p* < 0.05 was considered as significant.

Considering that EPAC1 is expressed in intestinal cell lines, we assessed if EPAC1 activation would lead to an increase in CFTR PM levels as shown in airway cells [9]. CFTR PM levels were assessed after EPAC1 activation in the CFBE and Caco-2 cell line, as it showed higher EPAC1 expression levels (Figure 4), using a cell surface biotinylation assay. First, cells were incubated with 007-AM to activate EPAC1 or DMSO (vehicle control). Next, cells were treated with biotin to label cell surface proteins. After protein extraction, biotinylated proteins were pulled down with streptavidin and CFTR was detected by WB (Figure 5A).

While results from CFBE cells reproduce the increase in wt-CFTR levels at the PM when EPAC1 is activated, compared to vehicle control (Figure 5B), the results for Caco-2 cells also show an increase in mature CFTR (band C) PM levels, which are even higher than in CFBE cells (Figure 5B).

Taken together, these results indicate that the EPAC1 stabilization pathway is active in intestinal cell lines. Evidence comes from airway and intestinal cells, even though it does not apply to the intestinal organoid model due to lack of EPAC1 expression.

## 4. Discussion

Regulation of CFTR at the PM is a complex process that involves multiple protein interaction partners and signaling pathways [5,6,7] and also depends on correct cytoskeletal organization to bring all these elements together [30]. One of the most relevant signaling pathways that regulate CFTR is cAMP signaling. In addition to the well-established role of cAMP in activating CFTR function through PKA activation [1], an additional role in the stabilization of CFTR at the PM in airway epithelial cells was more recently found [9]. The latter occurs through an EPAC1-dependent pathway triggered by cAMP concentrations higher than those required for PKA activation: EPAC1 translocates to the PM and interacts with CFTR through the adaptor protein NHERF1, promoting an increase in CFTR levels by decreasing endocytosis [9].

Better understanding of the processes that regulate the levels of CFTR at the PM is particularly relevant as even though the traffic of many CFTR mutants to the PM, including the most common F508del-CFTR, is rescuable by different strategies, a high endocytosis rate coupled with a reduction in recycling back to the PM cause a significant decrease in the half-life of the rescued mutant protein [31,32]. Therefore, CFTR PM stabilization can be targeted by a combinatorial therapeutical approach [33].

Here, we aimed at determining if the EPAC1-dependent CFTR stabilization, initially reported in airway cells, is tissue-specific. This pathway was assessed in intestinal models including intestinal organoids, one of the most used CF model systems. Ultimately, this approach enabled us to ascertain whether the organoid model could be a more physiological alternative to immortalized cell lines to study the EPAC1 pathway. The use of intestinal organoids in CF research has increased due to their physiological relevance, being derived from individuals with CF, and to the ability to correlate both basal CFTR function with disease severity and their response to drugs to predict clinical benefit, through the forskolin-induced swelling (FIS) assay [34].

We started by assessing the ability of 007-AM to activate EPAC1 by testing the induction of EPAC1 translocation to the PM (Figure 1) [8], through live-cell imaging in the CFBE bronchial cell line. Results showed translocation of EPAC1 from the cytoplasm to the PM only after treatment with 007-AM (Figure 1A) within the first 3 min of stimulation (Figure 1C). These results confirm that 007-AM effectively activates EPAC1 in airway cells.

Next, we used the FIS assay to assess if the EPAC1-dependent PM CFTR stabilization would lead to an increase in CFTR function (Figure 2), as the net CFTR activity depends not only on the gating of each individual channel, but also on the amount of protein at the PM [35]. Intestinal organoids with the D1152H/F508del CFTR genotype were used to avoid the pre-swelling effects that occur for wt-CFTR intestinal organoids [17]. The results show an increase in AUC for stimulation without Fsk (Figure 2A), in agreement with the reports of high residual function associated with the D1152H mutation [25]. In addition, EPAC1 activation with varying concentrations of 007-AM showed no effect on organoid swelling, with or without CFTR rescue by VX-661 (Figure 2B). These results indicate that the EPAC1 stabilization pathway does not occur in the intestinal organoid model.

We then postulate that failure of 007-AM in activating EPAC1 might be due to low expression of this protein in intestinal organoids. We thus assessed its expression levels by WB in intestinal organoids with four different CFTR genotypes (Figure 3). Indeed, the results show that there is no expression of EPAC1 in the intestinal organoid model, independently of CFTR mutation (Figure 3B). This reveals that the cause for absent EPAC1 stabilization effect is lack of this protein in intestinal organoids. There are reports of EPAC1 expression in intestinal cells [36,37,38], which even link EPAC1 to a role in intestinal Cl^−^ secretion in a mechanism independent of CFTR [37]. Organoids are derived from rectal tissue and this discrepancy in EPAC1 expression/signaling may derive from analysis of different intestinal regions in those studies. It is also possible that lack of expression in intestinal organoids is related to organoid preparation and culturing methods, supported by reports that, in mouse intestinal organoids, minor modifications of the culture medium generate different cell-type enriched organoids, leading to rewiring of the transcriptome and proteome [39].

Next, we assessed the EPAC1 stabilization pathway using intestinal cell line models starting with EPAC1 expression by WB (Figure 4). Results show EPAC1 expression in intestinal cell lines (Figure 4A) is in agreement with published work [36,37,38]. In comparison to airway cells, the NCM460 cell line shows reduced expression (down to 25%), while the Caco-2 cell line shows similar expression (Figure 4B). Lastly, the effect of EPAC1 activation on CFTR PM levels was assessed by cell surface biotinylation (Figure 5). Results for CFBE cells show an increase in CFTR PM levels under EPAC1 activation (Figure 5B), in agreement with the reported stabilizing effect [9]. In Caco-2 cells, EPAC1 activation also promotes an increase in PM CFTR levels (Figure 5B), demonstrating that the EPAC1 stabilization pathway of CFTR is relevant in this intestinal cell line.

In summary, our results reveal that EPAC1 is not expressed in the intestinal organoid model and thus the stabilization pathway does not apply in this model. Still, results from intestinal cell lines indicate that this pathway still occurs in intestinal-derived cells, confirming the EPAC1 stabilization pathway as a relevant mechanism to regulate CFTR PM stability (even if not in all biological models/cell types).

## Data Availability

Not applicable.
